# Modulation of Serotonin Receptors in Neurodevelopmental Disorders: Focus on 5-HT7 Receptor

**DOI:** 10.3390/molecules26113348

**Published:** 2021-06-02

**Authors:** Jieon Lee, Diana Avramets, Byungsun Jeon, Hyunah Choo

**Affiliations:** 1 Brain Science Institute, Korea Institute of Science and Technology, Seongbuk-gu, Seoul 02792, Korea; jieon@kist.re.kr (J.L.); avramets@kist.re.kr (D.A.); 2 Division of Bio-Medical Science & Technology, KIST School, Korea University of Science and Technology, Seoul 02792, Korea

**Keywords:** serotonin receptor, 5-HT_7_R, neurodevelopmental disorders, autism spectrum disorder, fragile X syndrome, Rett syndrome

## Abstract

Since neurodevelopmental disorders (NDDs) influence more than 3% of children worldwide, there has been intense investigation to understand the etiology of disorders and develop treatments. Although there are drugs such as aripiprazole, risperidone, and lurasidone, these medications are not cures for the disorders and can only help people feel better or alleviate their symptoms. Thus, it is required to discover therapeutic targets in order to find the ultimate treatments of neurodevelopmental disorders. It is suggested that abnormal neuronal morphology in the neurodevelopment process is a main cause of NDDs, in which the serotonergic system is emerging as playing a crucial role. From this point of view, we noticed the correlation between serotonin receptor subtype 7 (5-HT_7_R) and NDDs including autism spectrum disorder (ASD), fragile X syndrome (FXS), and Rett syndrome (RTT). 5-HT_7_R modulators improved altered behaviors in animal models and also affected neuronal morphology via the 5-HT_7_R/G_12_ signaling pathway. Through the investigation of recent studies, it is suggested that 5-HT_7_R could be a potential therapeutic target for the treatment of NDDs.

## 1. Introduction

Neurodevelopmental disorders (NDDs) are a group of disorders characterized by abnormal brain developmental processes which affect emotion, learning, cognition, and memory [1]. NDDs contain a wide range of disorders such as autism spectrum disorder (ASD), attention deficit hyperactivity disorder (ADHD), intellectual disabilities (IDs), and neurogenetic disorders [2,3,4]. Commonly, patients who are suffering NDDs have a comorbidity of two or more disorders. For instance, the majority of children with ADHD have language disabilities and considerable overlap exists in autism spectrum disorder and ADHD [5]. Although there are multiple causes of NDDs, which are social deprivation, genetic and metabolic diseases, nutrition, and infection, NDDs are typically associated with gene vulnerability, mutation, and environmental factors that influence the phenotype [6,7]. Besides, it has been proposed that environmental and genetic factors contribute to neuronal impairment and accordingly result in the occurrence of NDDs [8]. Neuronal processes, including outgrowth of dendrites and axons, are critical steps during early development [9] and alterations in the dendritic structure were found in multiple animal models of NDDs [10,11,12,13], which demonstrates a close relationship between neuronal morphology defects and NDDs. Despite efforts to recognize the fundamental etiology of NDDs, there are no cures for these disorders. Only a few FDA-approved drugs such as aripiprazole [14,15,16], risperidone [17,18], and lurasidone [19] have been applied to mitigate the symptoms. Several studies suggests that serotonin, one of the most studied neurotransmitters in our brain, plays a crucial role in the early neurodevelopmental stage [20]. We investigate the relationship between serotonin receptor subtype 7 (5-HT_7_R) as a therapeutic target and various NDDs including autism spectrum disorder (ASD), Rett syndrome (RTT), and fragile X syndrome (FXS).

5-HT_7_R is one of the serotonin receptor (5-HTR) subtypes and belongs to a family of G protein-coupled receptors (GPCRs) [21]. 5-HT_7_Rs are distributed in the various areas of the central nervous systems (CNS) such as the thalamus, hypothalamus, hippocampus, and cortex, and are involved in the regulation of sleep, circadian rhythm, learning, and memory and cognition [22,23]. It has been reported that 5-HT_7_R is coupled to G_s_ protein, resulting in an increase in intracellular cAMP, and also interacts with G_12_ protein which is one of the G_α_ subunits [24]. G_12_ protein interacts with various members of mammalian RhoGEFs, which activate Rho GTPases constituted with RhoA, Cdc42, and Rac1 [25]. Through the diverse studies, it is suggested that these Rho GTPases have an essential role in regulating cell morphology, actin cytoskeleton, neural branch dynamics, dendritic arbor, and neurite outgrowth [26,27]. It is identified that 5-HT_7_R mediated activation of G_12_ caused stimulation of RhoA and Cdc42 among the Rho GTPases, which resulted in activation of serum response element (SRE) regardless of Gs protein-induced activation of protein kinase A (PKA). In particular, RhoA and Cdc42 promote cell rounding and filopodia formation in the cell morphology. Accordingly, the stimulation of 5-HT_7_R in hippocampal neurons leads to an increase in neurite length, dendritic protrusions, and synaptic density, which is suppressed by SB269970, a selective 5-HT_7_R antagonist [24,28]. Also, it is suggested that cyclin-dependent kinase 5 (Cdk5), a signaling molecule known to regulate actin dynamic and stabilization in neurons, and Cdc42 are required to maintain 5-HT_7_R mediated spine formation, acting as downstream effectors of 5-HT_7_R. The inhibitors of Cdk5 and Cdc42 blocked or reduced dendritic spine formation and the number of dendritic spines, which are increased by 5-HT_7_R agonist [29]. There is another signaling pathway related to synaptic remodeling which includes 5-HT_7_R-mediated activation of matrix metalloproteinase 9 (MMP-9), which induces Cdc42 activation related to G_12_ signaling via hyaluronan receptor CD44 cleavage, resulting in neuronal outgrowth and dendritic spine elongation [30]. Interestingly, it has been reported that the expression of 5-HT_7_R and G_12_ were downregulated during later development, which restricted effects of 5-HT_7_R/G_12_ signaling on neuronal morphology to the early postnatal development stage [28]. These data are of great significance in that NDDs are primarily disorders of early development. Thus, the activation of the 5-HT_7_R/G_12_ signaling pathway appears to become an attractive therapeutic target for the treatment of NDDs.

The use of 5-HT_7_R agonists has been attempted in the treatment of several NDDs. Various 5-HT_7_R agonists exhibited the relief of hyperactivity, anxiety, and stereotypy and refined social ability in the ASD animal models [31] and, interestingly, FDA-approved antipsychotic drugs for ASD showed antagonistic activity against 5-HT_7_R [14,15,16,17,18,19]. Usually, agonists and antagonists of a receptor show opposite effects in in vivo study, while, in some case of 5-HT_7_R, agonists and antagonists have both positive effects on ASD, as well as antiamnestic effects in memory. The reason for the paradoxical effect is not revealed yet [32]. Meanwhile, though many studies suggest that various genetic mutations in a specific gene are represented as a major cause of RTT, there is no treatment for the RTT [33,34,35]. Current studies demonstrate the involvement of multiple factors for the manifestation of RTT, which are dysregulations of Rho GTPases and the serotonergic system [36,37]. Systemic administration of a 5-HT_7_R selective agonist improved anxiety profiles, environment-related exploratory behavior, and motor learning ability in the RTT animal model, in which the authors claimed that inactivation of Rho GTPases downstream effectors is reversed by the application of the 5-HT_7_R agonist [38,39,40,41]. FXS also occurs due to a genetic mutation, especially in the *Fmr1* gene, which is responsible for Fragile X mental retardation protein (FMRP) production, which leads to the overactivation of the signaling pathways via mGluR5 receptors and an increase in long-term depression (LTD) [42]. Additionally, it is reported that the stimulation of 5-HT_7_R not only corrected mGluR-mediated LTD but also improved repetitive behavior and social activity in the FXS animal model [43,44].

## 2. 5-HT_7_R/G_12_ Signaling Pathway

**Figure 1:**
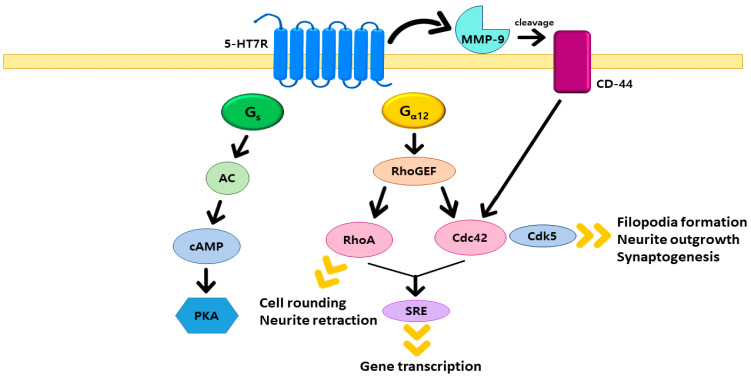
Schematic representations of 5-HT_7_R signaling pathways. Summary of the G_s_-mediated signaling pathway is shown on the left side and the G_12_-mediated signaling pathway which influences neuronal morphological alterations is depicted on the right side.

Among G_α_ subunits in heterotrimeric G proteins, the G_12_ subfamily consists of the G_12_ and G_13_ proteins, which were defined as the fourth class of G_α_ subunits [45,46]. G_12_ and G_13_ have been reported to bind to GPCRs that interact with various effectors such as Rho, p115RhoGEF, PDZ-RhoGEF, and leukemia-associated RhoGEF (LARG), which are known as the members of the mammalian RhoGEF proteins that activate small GTPase RhoA [47,48]. The Rho family of GTPases belongs to a subfamily of the Ras superfamily and contains 20 members in mammals [49]. The major members of the Rho family are Cdc42, Rac1, and RhoA, which are involved in regulating cell morphology, the actin cytoskeleton, neurite extension/retraction, and neurite outgrowth [50,51] (Figure 1). Li and co-workers investigated the involvement of Rho GTPases for neuronal branch dynamics and dendritic arbor growth in living *Xenopus* tadpoles [26]. The authors found that each of the three Rho GTPases had distinct effects on dendritic arbor development. RhoA activated by lysophosphatidic acid (LPA) inhibited dendritic branch extension, reducing dendritic arbor growth rate. Additionally, the growth rate of cells expressing negative RhoA treated with LPA was similar to that of the control neuron, which indicates that RhoA is responsible for the regulation of branch elongation. Conversely, Rac and Cdc42 did not affect the dendritic growth rate. Ruchhoeft and co-workers examined the effects of Rho GTPases on dendrite formation and growth cone morphology using *Xenopus* retinal ganglion cells (RGCs) expressed with wild-type, mutant RhoA, Rac1, and Cdc42 [27]. A loss of activities in Rac1 and Cdc42 lead to negative effects on dendrite formation in vivo. In the analysis of growth cone morphology, growth cones overexpressed with wt-Cdc42 had more filopodia and had larger back branches than controls, while RGCs expressing mutant Cdc42 showed the opposite effect. Interestingly, overexpressing wt-RhoA induced a decrease in the growth cone area. These findings that the diverse effectors of G_12_ manipulate neuronal morphology imply that the G_12_ signaling pathway plays an important role in regulating abnormal neuronal connectivity associated with neurodevelopmental disorders.

Recently, it has been reported that 5-HT_7_R is coupled not only to the Gs protein but also to the G_12_ protein [24]. Kvachnina and co-workers discovered the interactions between G_12_ protein and 5-HT_7_R by using [^35^S]GTPγS binding assay which determines the exchange of GDP-GTP for G_α_ subunits. Since it has been demonstrated that G_12_ protein regulated gene expression through transcriptional activation of serum response element (SRE) known as transcriptional control element [52,53], the authors investigated whether the 5-HT_7_R is involved in the activity of SRE, and identified that 5-HT_7_R-mediated activation of SRE occurred independently of G_s_ protein-induced activation of protein kinase A (PKA) Moreover, the researchers found that the Rho GTPases are engaged in SRE stimulation induced by 5-HT_7_R, of which RhoA and Cdc42, except for Rac1, were found to be activated by 5-HT_7_R, which are disclosed through the experiments measuring the ability of dominant-negative mutant of Rho GTPases to inhibit receptor-mediated SRE activation (Figure 1). Given that the Rho GTPases family is responsible for modulating neuronal morphology [26,27,50,51], the authors analyzed the morphology of NIH3T3 cells which were transiently transfected with 5-HT_7_R and found the expression of 5-HT_7_R lead to an increase of rounded and filopodia-bearing cells [24]. Also, the researchers discovered that RhoA regulates cell rounding, whereas Cdc42 regulates filopodia formation in cells transfected with RhoA and Cdc42 mutants and expressing 5-HT_7_R (Figure 1). To examine the role of the 5-HT_7_R in the regulation of neuronal morphology, the authors used dissociated hippocampal neurons and applied 5-HT_7_R agonist 5-CT to the neurons, which significantly increased the length of neurites; this effect was abolished when SB269970, a selective 5-HT_7_R antagonist, was administered, indicating that the activation of 5-HT_7_R/G_12_ signaling pathway contributes to neurite outgrowth. The group reported other results about the correlation of the 5-HT_7_R/G_12_ signaling pathway with neuronal morphology and function. Kobe and co-workers discovered that treatment of 5-CT, a 5-HT_7_R agonist, increased the number of dendritic protrusions and presynaptic marker synaptophysin which detects synaptic density [28]. The effects were diminished by the introduction of SB269970, implying that neuronal morphology is dependent on 5-HT_7_R. Additionally, the authors scrutinized the number of dendritic protrusions and the density of synapse in G_α12_ knockout (KO) neurons to analyze whether these morphogenic effects are mediated by 5-HT_7_R/G_12_. Both parameters in G_α12_ KO neurons were reduced compared with them in wild type, and the knockdown of 5-HT_7_R using siRNAs also decreased the number of dendritic protrusions and synaptic density. Speranza and co-workers reported that the application of 5-HT_7_R selective agonist LP-211 to striatal and cortical neurons increased neurite length, the number of dendritic protrusions, and the number of synaptic contacts, which is restrained by SB269970; these results are similar to the effects of treatment of 5-CT [29]. Furthermore, the authors revealed that cyclin-dependent kinase 5 (Cdk5) and Cdc42 have properties in the modulation of dendritic morphology and could be engaged in 5-HT_7_R mediated dendritic spine formation by analyzing the spine density of striatal neurons treated with Cdk5 inhibitor roscovitine and Cdc42 inhibitor ZCL 278. Both inhibitors abolished the effect induced by LP-211, which might suggest that Cdk5 and Cdc42 affect 5-HT_7_R mediated spine formation as downstream effectors. However, further studies will be needed to prove the contribution of 5-HT_7_R/Cdk5 to the receptor-mediated development of dendritic spines.

Bijata and co-workers reported that synaptic remodeling is associated with extracellular matrix (ECM) remodeling, which is uncovered through a signaling pathway including the 5-HT_7_R, matrix metalloproteinase 9 (MMP-9), the hyaluronan receptor CD44, and Cdc42 [30]. The authors discovered that stimulation of 5-HT_7_R by 5-CT and application of auto-activating MMP-9 (aaMMP-9) significantly increased the length of dendritic spines while no spine elongation was observed in cultures from MMP-9 KO and 5-HT_7_R KO mice. The researchers also found that the activation of 5-HT_7_R increased the activity of MMP-9, which is abolished by pretreatment of SB269970. These data suggest the involvement of 5-HT_7_R/MMP-9 signaling in dendritic spine alteration. To confirm the link between ECM and the above signaling, the authors focused on CD44, a receptor for a major ECM component, which connects the ECM to the intracellular signaling pathway related to the activation of the Cdc42. Silencing of CD44 leads to abrogation of 5-CT-induced increase in dendritic spines, and direct interaction and co-localization between 5-HT_7_R and CD44 have been identified with a fluorescence resonance energy transfer (FRET) based approach. Interestingly, the use of the Cdc42 inhibitor ZCL278, as well as CD44 KO, leads to suppression of dendritic spine elongation caused by the stimulation of 5-HT_7_R or treatment of aaMMP-9. These results suggest that an interaction between 5-HT_7_R and CD44 plays an important role in regulating Cdc42 activity concerning the spine morphology. In addition to these observations, the authors identified that MMP-9 can cleave the extracellular domain of CD44 in neurons, which is accomplished by the stimulation of 5-HT_7_R. Taken together, activated 5-HT_7_R results in the activation of MMP-9, which cleaves the extracellular domain of CD44, and then this cleavage, in turn, promotes morphological changes elicited 5-HT_7_R/Cdc42 signaling pathway (Figure 1).

It is important that the morphogenic effects of serotonin during developmental stages can control functions behaviorally related to neuronal networks in adulthood because NDDs mainly occur early in the development process [54]. Particularly, it has been reported that the 5-HT_7_R/G_12_ signaling pathway has effects on the regulation of various neuronal morphology during early development. The expression level of 5-HT_7_R and G_12_ protein in the mouse hippocampus at different postnatal developmental stages was examined by using quantitative RT-PCR, in which transcripts of 5-HT_7_R and G_12_ protein were highly expressed in the early stage and substantially diminished by almost up to ninefold in the later stage, but Gs protein was not affected during the development process. These expression patterns allow the effects of 5-HT_7_R/G_12_ signaling including dendritic morphogenesis, synaptogenesis, and functional plasticity of hippocampal networks to be applied only in the early stages of development [28]. Therefore, 5-HT_7_R/G_12_ signaling pathways may play an important role in regulating the onset of NDD, which occurs in the early development.

## 3. Autism Spectrum Disorder

Autism spectrum disorder (ASD) is the complex of neurodevelopmental conditions determined by several principal symptoms comprising stereotyped repetitive behavior patterns and restricted social interactions. Although a plethora of studies have been conducted since the ASD was discovered, the etiology and precise pathological mechanisms are still obscure. Current evidence suggests the involvement of both genetic and environmental factors in the occurrence and the course of the disorder. Gene polymorphism, epigenetic factors, perinatal complications, viral infections, exposure to toxic chemicals, and other aspects may participate in the onset and manifestation of autism in patients [55,56]. The major cause or consequence of ASD development is metabolic abnormalities and dysfunction of the various neurotransmitter systems in the brain including glutamate, gamma-aminobutyric acid (GABA), dopamine, acetylcholine, and serotonin [57]. In particular, at the beginning of the ASD investigation, the elevated serotonin levels in plasma and platelets were detected and considered to implicate the general pathophysiology [58,59]. Along with this, changes in densities of the serotonin receptors and transporters in the different brain areas were reported in autistic individuals [60,61]. Regarding 5-HT_7_R, one research using transmission disequilibrium test demonstrated an absence of correlation between *HTR7* gene polymorphism and ASD [62]. However, even though the dysfunction of 5-HT_7_R was not reported in patients with autism, this type of receptor has some indirect connections to be a plausible target for the treatment of ASD or at least improving symptoms and behavioral condition [63]. Wu and co-workers reported that the application of deep brain stimulation with the administration of 8-OH DPAT, a 5-HT_1A_R/5-HT_7_R agonist, remarkably alleviated hyperactivity and anxiety profiles and refined sociability in the valproate (VPA)-induced rat ASD model [31] (Table 1). Moreover, the authors observed that the expression of *N*-methyl-D-aspartate receptor (NMDA) and GABA receptor subunits were reduced, resulting in normalized excitatory and inhibitory processes in neural circuits. Wang and co-workers revealed that 8-OH DPAT treatment consistently rescued social behavior and fear memory in VPA-induced rats and, besides, improved presynaptic excitatory transduction [64]. However, the authors fail to claim whether these mechanisms are regulated by 5-HT_1A_R or 5-HT_7_R. Canal and co-workers reported that amino tetralin derivative (+)-5-FPT showed high affinity and partial agonism against 5-HT_7_/5-HT_1A_Rs and reduced stereotypy in three heterogeneous mouse models as well as increased social interaction [65] (Table 1). Moreover, based on thorough pharmacokinetic studies, the authors claimed that (+)-5-FPT appears to be a potent lead to treat ASD and related symptoms. Besides (+)-5-FPT, several chemical drugs are good examples in proving the possible involvement of 5-HT_7_Rs in the modulation of ASD. Aripiprazole, an FDA-approved antipsychotic drug for autism, demonstrates notable affinity to different dopamine and serotonin receptors, including antagonistic effects on 5-HT_7_R as well. This therapeutic agent efficiently targets irritability in patients diagnosed with autism, Asperger’s syndrome, schizophrenia, and other neurodevelopmental disorders [14,15,16,66]. Consistently, other pharmacological treatments for ASD, such as risperidone and lurasidone, which are used to alleviate aggressive behavior in patients, show antagonistic activity against 5-HT_7_Rs [17,18,19,66] (Table 1). Lacivita and co-workers recently reported the development of several arylpiperazine derivatives, **1**, **2**, and **3**, which demonstrated double 5-HT_7_R/5-HT_1A_R agonistic activity or combined 5-HT_7_R/5-HT_1A_R activating/5-HT_2A_R antagonistic properties [67] (Table 1). Few of these molecules showed high metabolic stability, drug-like properties, and functional activity for corresponding signaling pathways, potentially modulating the ASD etiology and progress, revealing a novel approach in the drug discovery for this disorder. Apart from that, many researchers have suggested that changes in neurodevelopmental patterns such as increased neuronal proliferation, defects in neuronal migration, abnormal neurite outgrowth, and dysregulation of synaptic plasticity could be responsible for ASD [68,69,70]. Lin and co-workers recently surveyed the involvement of small GTPases and their downstream effectors’ pathways in the mechanisms of neurodevelopmental disorders [71]. These specific signaling pathways have direct interconnections with other factors, responsible for cell proliferation, motility, migration, and, subsequently, for the maintenance of normal neuronal morphology [72]. Notably, a widely used ASD-like *Shank3*-deficient mouse model displayed impaired Rac1/PAK/cofilin signaling and decreased F-actin expression in the cortex, while inhibition of cofilin rescued actin filament levels and markedly improved behavioral patterns in ASD-like mice [73]. Another study performed on *TAOK2*-knockout mice, which demonstrate cognitive dysfunction, revealed aberrant dendritic morphology and synapse formation through dysregulation of RhoA signaling in this animal model [74]. Moreover, the connectivity between 5-HT_7_R and G_12_ signaling networks and modulation of neurite growth and synapse plasticity has been recently studied [24,28]. Consequently, as well as RhoA-mediated signaling pathways modulate the regulation of the actin cytoskeleton reorganization, impairments in this network may lead to aberrant neurite architecture, resulting in dysfunction of synaptic signal transmission. Apart from that, few studies have reported possible molecular mechanisms involved in the regulation of neuron connectivity upon the developmental process. Speranza and co-workers demonstrated that the treatment of neuron cell cultures with 5-HT_7_R selective agonist LP-211 promoted neurite growth via cell division cycle 42 (Cdc42), *mammalian target of rapamycin* (mTOR), cyclin-dependent kinase 5 (Cdk5), and extracellular signal-regulated kinase (ERK) molecular network [75,76] (Table 1). The research group further showed that continuous application of LP-211 leads to a prominent increase in the number of dendritic spines density and synaptic contacts [29]. Taken together, 5-HT_7_R may play a crucial role in further investigation of mechanisms and the development of treatment strategies for ASD and other disorders.

## 4. Fragile X Syndrome (FXS)

Fragile X syndrome (FXS) is a common neurodevelopmental disorder characterized by strong intellectual disability, and usually associated with autism spectrum disorder [79]. People affected with this syndrome bear a genetic mutation in the Fragile X mental retardation 1 (*Fmr1*) gene, which is responsible for Fragile X mental retardation protein (FMRP) production [79]. FMRP is an mRNA-binding protein that plays an important role in the negative regulation of protein synthesis, and specifically in brain changes in the levels of FMRP that contribute to cognitive dysfunction. In particular, along with the available evidence, the metabotropic glutamate receptors (mGluR) theory of FXS has been developed. According to this notion, the loss of Fmr1 causes abnormal protein synthesis as well as overactivation of signaling via mGluR5 receptors, increases long-term depression (LTD) and, subsequently, induces aberrant synaptic plasticity [42,80]. Recent studies demonstrated that the stimulation of serotonin receptors, utilizing agonist agents, may modulate the mGluR signaling pathway and rescue impaired features in Fragile X syndrome models. Lim and co-workers reported that the activation of the 5-HT2BR boosted Ras-phosphoinositide 3-kinases-RAC-alpha serine/threonine-protein kinase (Ras–PI3K/Akt) signaling pathway improved glutamate receptor 1(GluA1)-mediated synaptic plasticity, and showed beneficial effects on the learning ability of FXS mice model [81]. Besides, Costa and coauthors reported that the stimulation of 5-HT7R by non-selective agonists, such as serotonin and 8-OH DPAT, decreased mGluR-mediated LTD and prevented internalization of α-amino-3-hydroxy-5-methyl-4-isoxazolepropionic acid (AMPA) receptors in the hippocampal tissue slices [82] (Table 1). Since Fmr1-knock out mice exhibit sustained upregulation of mGluR-mediated LTD and a reduced density of AMPA receptors, the administration of 8-OH DPAT consistently reversed this pathological condition to the normal level [82]. Furthermore, the authors scrutinized the effects of LP-211, a 5-HT7R selective agonist, on LTD and confirmed their previous results [43] (Table 1). The further study was aimed at designing novel 5-HT7R selective agonist compounds with improved pharmacokinetic parameters and higher efficacy. Based on the LP-211 chemical structure, Costa and co-workers developed a BA-10 compound that showed greater metabolic stability and higher affinity to 5-HT7R [44] (Table 2). Both LP-211 and BA-10 displayed effective correction of mGluR-LTD in wild-type and Fmr1-deficient mice, demonstrating the potential to modulate the impairment in synaptic plasticity in FXS. As far as 5-HT7Rs are coupled with Gs subunit, which activates adenylate cyclase, and several studies [83,84] reported an aberrant cAMP metabolism in patients with FXS, it was suggested that an impaired cAMP-mediated signaling pathway may be involved in the exaggerated generation of LTD. Costa and co-workers further discovered that treatment with forskolin and pituitary adenylate cyclase-activating polypeptide (PACAP), which are supposed to be stimulators of adenylate cyclase, completely replicated the effects of LP-211 as expected, while simultaneous application of the 5-HT7R agonist with adenylate cyclase or protein kinase A (PKA) blockers prevented the LTD reversal to the normal rate [82] (Table 2). However, there is controversial evidence claiming that impairment in cAMP metabolism may be resulted not from aberrations in the signaling via 5-HT7R, but D1 dopamine receptors [83]. Nevertheless, in vivo administration of LP-211 to young Fmr1-knock out mice significantly improved stereotypic behavior and recognition memory [83]. Taking all the following evidence into consideration, there is a strong demand for the development of novel 5-HT7R agonists with improved pharmacokinetic properties and activity. Armstrong and co-workers recently reported that treatment with the orally operative aminotetraline compound (+)-5-FPT [65], which is a partial agonist of 5-HT1A, 5-HT2C, and 5-HT7 receptors, leads to significant improvement in the phenotypic condition [78] (Table 1). In particular, it considerably mitigated repetitive behavior, markedly reduced the occurrence of lethal audiogenic seizures, which are typical for Fmr1-KO condition, and elevated the social activity both in wild-type and FXS transgenic mice [78]. As well as 5-HT_1A_R, 5-HT_2C_R was shown to be involved in the activation of phospholipase C beta (PLCβ), protein kinase C (PKC), and, as a consequence, the mitogen-activated protein kinase/extracellular signal-regulated kinase (ERK/MAPK) signaling pathway, which is essential for normal cell functioning, and its alteration is detected in various neurological disorders [85,86,87]. Another research group also suggested that this pathway may be modulated by 5-HT2CR coupling with G12/13 subunits, which explains the beneficial effects of 5-HT2CR activation [88]. However, the design and trials of compounds selective to several receptors just alleviate the condition but cannot elicit the precise molecular mechanisms of particular disorders. By exploiting validated scaffolds in bioactive compounds, Lacivita and co-workers synthesized and examined a variety of long-chain arylpiperazine compounds with biased selectivity to 5-HT7R. Among all developed compounds, the authors claimed that a compound especially showed drug-like properties, manifesting high affinity, distinctive selectivity to 5-HT7 receptor type, upgraded metabolic stability, and, besides, it significantly mitigated stereotypic behavior of FXS model mice [89] (Table 2).

## 5. Rett Syndrome

Rett syndrome (RTT) is a severe neurodevelopmental disorder, the second most common cause of mental retardation in females, which is usually indicated by such symptoms as breathing dysfunction, loss of coordination, abnormal eye and hand movements, seizures, aberrant sleeping behavior, and cognitive disabilities [90,91]. The prime cause of the syndrome is various genetic mutations in *methyl CpG binding protein 2* gene (*MeCP2*) on the X chromosome that commonly lead to more than 90% of overall cases, depending on the locus to the maintenance of phenotypic variability of the RTT [33]. Other atypical disorder occurrences are connected to abnormalities in other genes such as *cyclin-dependent kinase-like 5* (*CDKL5)*, *forkhead box G1* (*FOXG1)*, *WD repeat domain 45* (*WDR45),* or *syntaxin binding protein 1 (STXBP1)* [34,35]. Currently, there is no known drug for the RTT, therefore the amelioration of symptoms and particular conditions can become a solution for patients diagnosed with RTT. Although Collins and co-workers demonstrated that restoring the *MECP2* function can normalize function in MeCP2-null mice, manipulating the *MECP2* gene as potential gene therapy may lead to undesirable consequences, since it was shown that overexpression of this gene led to neurological defects [92]. Thus, targeting for MeCP2 downstream effectors and other signaling pathways may be taken into consideration. Recent studies have shown the involvement of different factors, including brain-derived neurotrophic factor (BDNF), insulin-like growth factor 1 (IGF-1), RhoA family of GTPases, and neurotransmitter systems, in the maintenance of the major clinical manifestations of RTT. Additionally, some research groups focused on the dysregulation and participation of the serotonergic system in animal models of RTT [37,38,39,40,93,94,95,96,97,98]. Especially, Abdala and coauthors surveyed recent studies of 5-HT_1A_R agonists for the syndrome and addressed that selective 5-HT_1A_R agonists could be a potential breakthrough to cure the disorder [94]. The group also found that the administration of 8-OH DPAT, a 5-HT_1A_R agonist, decreased the number of apneas and reduced the irregularity of the respiratory cycle in *MeCP2*-deficient mice, the model of RTT, even though the authors failed to address its mode of action [77] (Table 1). Further, Levitt and co-workers tested 5-HT_1A_R selective agonist F15599 in MeCP2-deficient mice and MeCP2-null mice, and displayed that F15599 helps to improve respiration via the activation of G-protein coupled inwardly, rectifying potassium channels (GIRK) without influencing glutamate release [93] (Table 3). Abdala and co-workers conveyed their previous concept with clinically approved saritozan, a 5-HT_1A_R agonist and a dopamine D2-like agonist/partial agonist, and confirmed the positive impact of 5-HT_1A_R activation on the alleviation of respiratory dysfunction in RTT established model mice [95] (Table 3). The mechanism of 5-HT_1A_R agonists for the disorder is still unclear but a study with F15599 helps to consider that the activation of GIRK by F15599 leads to discouraging the overactivation of the expiratory neurons, resulting in improved respiration [93]. It is noteworthy that within the aforementioned disorders and other reviewed topics relevant to brain functioning, an interplay between 5-HT_1A_R and 5-HT_7_R was observed [99,100]. These serotonin receptor types belong to different GPCR classes and canonically couple to diverse Gα subunits with opposite effects, such as Gi in the case of 5-HT_1A_R and Gs for 5-HT_7_R, resulting in adverse modulation of adenylate cyclase and affecting antagonistically cAMP concentration in the cell [101,102]. The recent studies showed that established facts about 5-HT_1A_R signaling pathways are actually ambiguous and that it also may control other downstream effectors, including ERK/MAPK, Pi3K-Akt signaling pathways, and cation channels, as well as 5-HT_7_R [103,104,105]. Besides, the effects of the 5-HT_1A_R activation on PLC were demonstrated that triggered subsequent modulation of PKC and caused positive effects on synaptogenesis [106]. Aforesaid networks are known to be implicated in the regulation of the actin cytoskeleton reorganization and, thus, various cell functions and synaptic plasticity [104]. Moreover, it was demonstrated that, in the hippocampus, 5-HT_1A_R targets highly expressed adenylate cyclase II which leads to the increase of the cAMP cellular level, showing that consequences of this receptor activation depend on the particular agonist and brain structure where it was affected [107,108]. Apart from that, in vitro and in vivo studies revealed specific interactions between 5-HT_1A_R and 5-HT_7_R, resulting in heterodimers formation and suggesting that 5-HT_7_R plays a dominant role in the complex and inhibits Gi activation via 5-HT_1A_R and, subsequently, regulates its downstream pathways [109]. As a result, all mentioned evidence may shed light on a complex interplay between these serotonin receptors and, to a certain extent, explain controversial findings of their involvement in the alleviation of neurodevelopmental pathological conditions. Apart from 5-HT_1A_R, Vogelgesang and co-workers reported that expression levels of the 5-HT_5b_R were markedly elevated in MeCP2-knockout mice [97]. Subsequent research revealed that additional knockout of 5-HT_5b_R significantly improved respiratory pattern and slightly increased the lifespan of mice with RTT phenotype [98]. This phenomenon may be explained by the hypothesis that intracellular 5-HT_5b_ receptors via coupling with the Gi subunit decreases the total level of cAMP and, thus, impairs the whole signaling network in the cell [97]. Interestingly, 8-OH DPAT known as a 5-HT_1A_R agonist showed activating effects on another kind of serotonin receptor family 5-HT_7_R, so the effects of treatment may be relevant to this receptor-type stimulation as well [96]. In addition, recent studies indicated 5-HT_7_R and corresponding coupled sgnaling pathways are linked to the course of the RTT [36,37,38,39,40,41]. De Filippis and co-workers observed that the density of 5-HT_7_R in cortical and hippocampal areas was lowered in Mecp2-308 male mice; an RTT model and systemic administration of 5-HT_7_R selective agonist LP-211 was able to relieve RTT-related defective symptoms including anxiety profiles, environment-related exploratory behavior, and motor learning ability [38] (Table 1). The authors also demonstrated that inactivation of Rho GTPases downstream effectors, such as cofilin and the p21-activated kinase family, which regulate actin cytoskeleton polymerization, is increased in RTT mice, and the introduction of LP-211 restored their activities via inhibiting the phosphorylation. In addition, LP-211 rescued the phosphorylation levels of ribosomal S6 protein, which is crucial in the regulation of translation in model animals (Table 1). The results were further evaluated by pursuing the same experiments on Mecp2-308 heterozygous female mice, a female-based Rett syndrome model [39]. After seven-day treatment with LP-211, these RTT mice exhibited refined phenotypic alterations, locomotor response, and synapse potentiation in comparison with vehicle-treated mutant animals, and also data supporting that 5-HT_7_R agonist treatment increases levels of phosphorylated S6 protein is consistent with previous research. It is noteworthy that the seven-day administration of LP-211 demonstrates a long-lasting effect in Mecp2-308 heterozygous female mice [39] (Table 1). Apart from that, Valenti and co-workers postulated that the activation of RhoGTPases via 5-HT_7_R recovers mitochondrial dysfunction in Mecp2-308 and MeCP2-Bird mice. Notably, complete rescue of electron transport chain (ETC) complexes activity and whole-brain ATP levels restoration was achieved in both RTT mice models after LP-211 systemic administration. Furthermore, LP-211 treatment also prevented the overproduction of reactive oxygen species in brain tissue, which was detected in MeCP2-deficient mice [40] (Table 1). As aforementioned, rare cases of RTT can be caused not by *MECP2* gene mutations, but other ones such as *cyclin-dependent kinase-like 5* genes (*CDKL5*). Vigli and co-workers pursued experiments on CDKL5-knockout mice that represent the set of the symptoms specific for CDKL5 deficiency disorder (CDD), which has a high similarity to the classical RTT [41]. Stimulation of 5-HT_7_R by selective agonist LP-211 slightly reversed the pathological condition to wild type level resulting in decreased pre-pulsed inhibition, normalized activation of ribosomal S6 protein, and rescued mitochondrial function [41] (Table 1). All the observed findings lead us to consider 5-HT_7_ receptors as potential targets to relieve symptoms in patients diagnosed with the RTT. However, further investigations are compulsory to clarify molecular mechanisms of particular disorders more specifically and to find new therapeutic agents.

## 6. Conclusions

In this review, we focused on the association of NDDs and 5-HT_7_R as a therapeutic drug target for the treatment of NDDs, and have explored ASD, RTT, and FXS, which are the most representative of various NDDs. 78Though several therapeutic agents for ASD including aripiprazole, risperidone, and lurasidone showed antagonism toward 5-HT_7_R, 8-OH-DPAT, (+)-5-FPT, and the most recently developed arylpiperazine derivatives which acted as agonists against 5-HT_7_R rescued social behavior, fear memory, and stereotypy in ASD mice model. Furthermore, it is observed that small Rho GTPases have a direct or indirect connection with neuronal morphology in ASD mice. 5-HT_7_R agonists displayed beneficial effects such as correction of mGluR-LTD, improved stereotypy, recognition memory, reduced the occurrence of lethal audiogenic seizures, and elevated social activity in various FXS mice models. Some studies demonstrated the potential to regulate the impairment in synaptic plasticity in FXS. Since it is reported that the serotonergic system is one of the manifestations of RTT, many research groups pay attention to 5-HTRs. Among them, a correlation has recently been reported between 5-HT_7_R and RTT, which is a lower density of 5-HT_7_R in RTT mice and refined phenotypic conditions by 5-HT_7_R agonist in RTT mice. Through the studies regarding 5-HT_7_R/G_12_ signaling pathways, it is identified that activation of 5-HT_7_R by diverse agonists leads to alterations of neuronal morphology such as length of neurites, dendritic protrusions, and density of synapse, which affect neurodevelopment. Also, it is revealed that the small Rho GTPases are responsible for modulating neuronal morphology in the 5-HT_7_R/G_12_ signaling pathways. Taken together, these findings that the stimulation of 5-HT_7_R via G_12_ signaling has direct or indirect neuromorphological effects on various NDDs indicate that modulators of 5-HT_7_R/G_12_ can be promising therapeutic agents for multiple NDDs.

## Figures and Tables

**Table 1:** Pharmacological agents which have potential effects on ASD and neurodevelopmental disorders treatment.

Names	Structures	Targets	Effects
8-OH DPAT	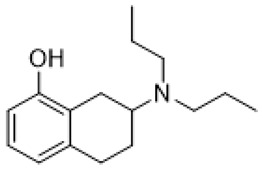	5-HT_1A_R/5-HT_7_R agonist	decreased mGluR-mediated LTD, prevented internalization of AMPA receptors [43]; normalization of respiratory function [77]
(+)-5-FPT	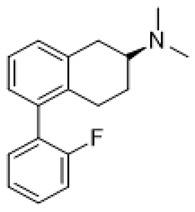	5-HT_1A_R/5-HT_2C_R/5-HT_7_R agonist	reduction of stereotypic behavior, social activity increase [65]; reduced the number of audiogenic seizures [78]
aripiprazole	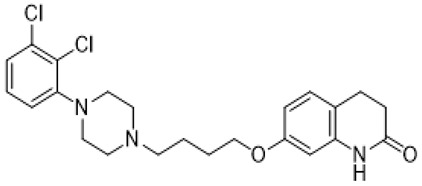	partial 5-HT_1A_R/ HT_2A_R/5-HT_2C_R/5-HT_7_R/D_1_R/D_2_R/D_3_R/D_4_R/D_5_R agonist; 5-HT_1B_R/5-HT_1D_R/5-HT_2A_R/5-HT_2C_R/5-HT_3A_R/HT_6_R/5-HT_7_R/D_1_R/D_2_R/D_3_R/ D_4_R/D_5_R/some alpha adrenergic and histamine receptors antagonist	irritability amelioration [14,15,66]
risperidone	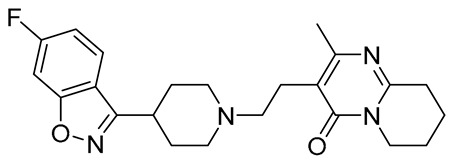	5-HT_1A_/5-HT_1D_R/5-HT_2A_R/5-HT_2C_R/5-HT_7_R/D_1_R/D_2_R/some alpha adrenergenic and histamine receptors antagonist	irritability and aggressive behavior amelioration [17,18,66]
lurasidone	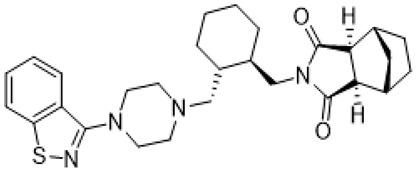	5-HT_1A_R/5-HT_2A_R/5-HT_7_/D_2_R/ some alpha adrenergic receptors antagonist; 5-HT_1A_R partial agonist	irritability and aggressive behavior amelioration [19,66]
**1**	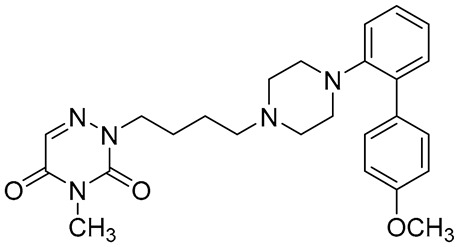	5-HT_1A_R/5-HT_7_R agonist	metabolically stable and have suitable CNS druglike properties [67]
**2**	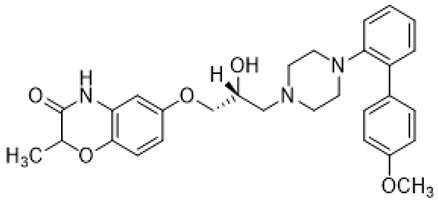	5-HT_1A_R/5-HT_7_R agonist	metabolically stable and have suitable CNS druglike properties [67]
**3**	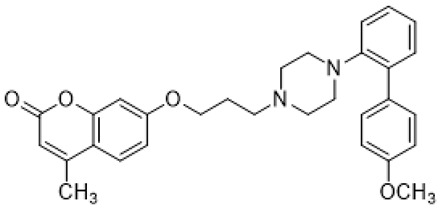	5-HT_1A_R/5-HT_7_R agonist; 5-HT_2B_R antagonist	metabolically stable and have suitable CNS druglike properties [67]
LP-211	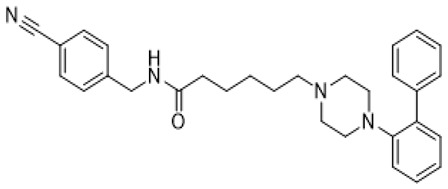	5-HT_7_R agonist; affinity to 5-HT_1A_R/D_2_R	neurite growth promotion (increased the number of dendritic spines and synaptic connections) [54,68,69]; decreased mGluR-mediated LTD, prevented internalization of AMPA receptors [43,44]; reduction of stereotypic behavior, improvement of recognition memory [38,39]; anxiety alleviation, exploratory behavior and learning ability improvement [38,39]; normalization of mitochondrial ETC function [40,41]

**Table 2:** Pharmacological agents which have potential effects on FXS treatment.

Names	Structures	Targets	Effects
BA-10	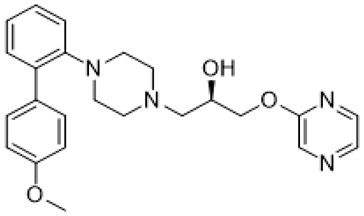	5-HT_7_R agonist	decreased mGluR-mediated LTD [44]
Forskolin	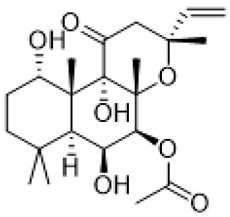	adenylate cyclase stimulation	decreased mGluR-mediated LTD [82]
PACAP	a protein encoded by *ADCYAP1* gene	adenylate cyclase stimulation	decreased mGluR-mediated LTD [82]
**4**	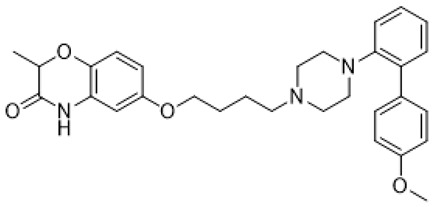	5-HT_7_R agonist	reduction of stereotypic behavior [89]

**Table 3:** Pharmacological agents which have potential effects on RTT treatment.

Names	Structures	Targets	Effects
F15599	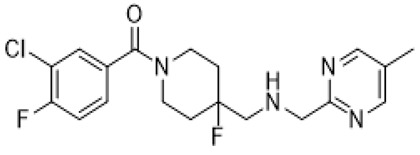	5-HT_1A_R agonist	normalization of respiratory function [93]
sarizotan	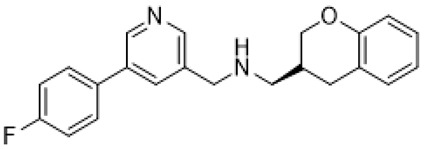	5-HT_1A_R agonist, affinity to D_2_R/D_3_R/D_4_R	normalization of respiratory function [66,95]
